# Optimization of macroelement concentrations, pH and osmolarity for triacylglycerol accumulation in *Rhodococcus opacus* strain PD630

**DOI:** 10.1186/2191-0855-3-38

**Published:** 2013-07-15

**Authors:** Helge Jans Janßen, Mohammad H A Ibrahim, Daniel Bröker, Alexander Steinbüchel

**Affiliations:** 1 Institut für Molekulare Mikrobiologie und Biotechnologie, Westfälische Wilhelms-Universität Münster, Corrensstrasse 3, D-48149, Münster, Germany; 2 Environmental Sciences Department, King Abdulaziz University, Jeddah, Saudi Arabia; 3 Natural and Microbial Products Chemistry Department, Pharmaceutical & Drugs Industries Research Division, National Research Centre, Dokki, Egypt

**Keywords:** Biodiesel, Biofuels, Lipids, Rhodococcus opacus, Triacylglycerols

## Abstract

The refinement of biodiesel or renewable diesel from bacterial lipids has a great potential to make a contribution for energy production in the future. This study provides new data concerning suitable nutrient concentrations for cultivation of the Gram-positive *Rhodococcus opacus* PD630, which is able to accumulate large amounts of lipids during nitrogen limitation. Enhanced concentrations of magnesium have been shown to increase the final optical density and the lipid content of the cells. Elevated phosphate concentrations slowed down the onset of the accumulation phase, without a clear effect on the final optical density and the cell’s lipid content. A robust growth of *R. opacus* was possible in the presence of ammonium concentrations of up to 1.4 g l^-1^ and sucrose concentrations of up to 240 g l^-1^, with an optimum regarding growth and lipid storage observed in the range of 0.2 to 0.4 g l^-1^ ammonium and 20 to 40 g l^-1^ sucrose, respectively. Moreover, *R. opacus* showed tolerance to high salt concentrations.

## Introduction

Due to the expected depletion of fossil fuel sources in the near future, increasing attention is paid to the development of alternative sources of energy. Bioethanol and biodiesel (fatty acid alkyl ester, FAAE) are among others the most interesting organic compounds, currently comprising about 90% of the biofuel market (Antoni et al. 2007;Uthoff et al. 2009). Today the industrial production of biodiesel is restricted to the transesterification of fatty acids derived from oleaginous plants like rapeseed, oil palm and soya or from animal fats (Luque et al. 2008). Because of the low price for methanol, fatty acid methyl esters are mostly produced (Al-Zuhair 2007), but other short-chain length alcohols can be used as well for the production of biodiesel with altered properties (Röttig et al. 2010). The transesterification can be done either by chemical or enzymatic catalysis or *in vivo*, by the use of microbial cells (Adamczak et al. 2009). Alternatively, oils or fats can be hydrotreated (hydrogen de-oxygenation) to produce renewable diesel or jet fuel. Renewable diesel is higher quality fuel component compared to biodiesel. Renewable diesel consists of paraffinic hydrocarbons alike fossil diesel and is thus fully compatible with existing fuel distribution systems and engines. Despite their application for biodiesel production on industrial scale, the use of plant derived fatty acids cannot satisfy the future energy demand because the agricultural area needed for sufficient production of triacylglycerols (TAGs) for biodiesel would have to be much larger than the currently cultivated area (Rude and Schirmer 2009). Furthermore, the use of these agricultural areas for biodiesel production competes with their use for food production (McDonald et al. 2009).

One possible alternative to overcome the limits of plant TAG-derived biodiesel production is to convert abundant waste and residue materials, e.g. lignocellulosic materials, to oils by using oleaginous bacteria like the soil bacterium *Rhodococcus opacus* strain PD630 for the production of TAGs (Alvarez and Steinbüchel 2002;Ratledge 2010;Kurosawa et al. 2010). This bacterium, belonging to the order of the *Actinomycetales*, was shown to accumulate TAG up to 76% (*w/w*) of the cell dry weight when growing on gluconate, and up to 87% when growing on oleic acid as sole carbon source (Alvarez et al. 1996). Voss and Steinbüchel (2001) reported the production of TAG in 500 liter scale by fed batch fermentation of *R. opacus*, yielding a cell density of 18.4 g l^-1^ consisting of up to 38.4% TAG. Recent investigations have led to an optimized batch process, yielding a cell density of 77.6 g l^-1^ with still 38.4% TAG in a low volume Sixfors bioreactor system (Kurosawa et al. 2010). However, a scale up of this process was not done, yet. An interesting finding was that *R. opacus* performed best in a medium containing 240 g l^-1^ glucose and 13.45 g l^-1^ (NH_4_)_2_SO_4_, even though a long *lag*-phase occurred.

Besides the capability of TAG production, many species of the genus *Rhodococcus* are known to have a wide substrate utilization range which enables them to grow on a variety of alkanes and aromatic compounds (Finnerty 1992). Due to the production of surfactants like glycolipids and exopolysaccharides (Lang and Philip 1998), their cellular surface is rather hydrophobic which makes the bacteria quite tolerant to organic solvents (Na et al. 2005Hori et al. 2009). Accordingly *R. opacus* strain B-4 has been shown to perform the conversion of water-immiscible chemicals much better than the rather hydrophilic bacterium *Escherichia coli* (Hamada et al. 2009).

The objective of our study was to investigate the nutrient requirements for TAG production with *R. opacus*. Besides different concentrations of the macroelements, the effect of high concentrations of magnesium ions in the medium was investigated because these ions are needed as cofactor for the acetyl-CoA carboxylase, the enzyme that is responsible for the first step in fatty acid biosynthesis (Cronan and Waldrop 2002). The results of this study will be helpful for the optimization of previously reported fermentation processes.

## Materials and methods

### Strain and media

In this study, *R. opacus* strain PD630 (DSMZ 44193; Alvarez et al. 1996) was investigated with regard to the production of lipids. All cultivations were carried out in mineral salts medium (Schlegel et al. 1961) with SL6 (Pfennig 1974) as source of trace elements. The pH was adjusted to 7.5 by the addition of NaOH. Sucrose was used as carbon source, normally in a concentration of 40 g l^-1^. For solid media 15 g l^-1^ Bacto agar were added to the medium prior to autoclaving. However, as specified in the text, in many experiments this basic medium was modified.

Concentrations of nitrogen and phosphorus are indicated as concentrations of ammonium and phosphorus, not of the corresponding salts. For example 3.4 g l^-1^ phosphorus results from the addition of 1.5 g l^-1^ KH_2_PO_4_ and 4.5 g l^-1^ Na_2_HPO_4_ x 2H_2_O. For reduced or elevated phosphorus concentrations, the ratio of the salts was kept the same.

For the calculation of the osmotic pressure (= osmolarity) of the growth medium it was assumed that all salts dissociate completely. Though this is not absolutely exact, it is sufficient for the conclusions that are drawn in this study.

### Cultivation in Erlenmeyer flasks

Precultures of 20 ml medium in 100 ml Erlenmeyer flasks equipped with baffles were inoculated with cells from a single colony of *R. opacus*, grown on solid medium, and were incubated for 24 h. Flask experiments were done with 50 ml medium in 250 ml Erlenmeyer flasks equipped with baffles and inoculated from the preculture to an optical density (OD) at 600 nm of 0.05. All flasks were incubated on a horizontal rotary shaker at 30°C and agitated at 105 rpm.

For the cultivation with reduced phosphate concentrations, bromothymol blue was added to the medium to a final concentration of 1 mg l^-1^. By the addition of 1 M NaOH, the pH values of all flasks with limited phosphate were adjusted to that of the control cultures. This was done every 10 hours by comparing the color of bromothymol blue.

Growth was monitored by measuring the OD at 600 nm with a GENESYS 20 spectrophotometer (Thermo Fischer Scientific, Schwerte, Germany). Every experiment represents the mean of at least two cultivations.

### Analysis of the cellular fatty acid contents

The contents of fatty acids of the cells was analyzed by methanolysis of the lipid-containing cells and subsequent gas chromatographic analysis of the resulting fatty acid methyl esters (Brandl et al. 1988;Wältermann et al. 2000). Fatty acids of a known amount, handled in the same way, were used as standards for identification and quantification.

For determination of the fatty acid content, about 7 mg lyophilized cells were incubated at 100°C for 4 h in 2 ml chloroform and 2 ml methanol, suspended with 15% (*v/v*) sulfuric acid. After extraction with 1 ml water, the organic phase was taken, and 2 μl of it were analyzed after 1:20 split injection on an Agilent 6850 gas chromatograph (Agilent Technologies, Waldbronn, Germany) with a BP21 capillary column (50 m x 0.22 mm, film thickness 250 nm, SGE Analytical Science, Darmstadt, Germany). Hydrogen was used as carrier gas with a constant flow of 0.6 ml min^-1^. The injector and the flame-ionization detector (Agilent Technologies) had temperatures of 250°C and 275°C, respectively. During the run, the temperature was altered according to the following schedule: 120°C for the first 5 min, for 20 min an increase by 3°C min^-1^ to 180°C, for 4 min an increase by 10°C min^-1^ to 220°C which was then kept for 31 min.

## Results

### Limitation of certain nutrients

**Figure 1:**
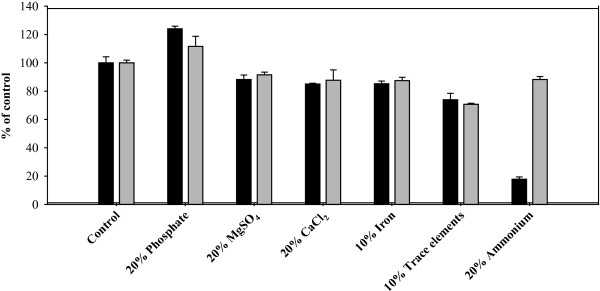
**Growth and fatty acid content with different nutrient limitations.** Cells of *R. opacus* were grown in mineral salt medium with 40 g l^-1^ sucrose as carbon source, but with different components of the medium reduced to 10% or 20% of their standard amount. After cultivation the final OD (black bars) and fatty acid content (grey bars) were determined and were illustrated as percentages of the control culture that was grown under standard conditions. Error bars represent the deviations of at least two independent cultivations.

The limitation of certain compounds in the growth medium was done to identify the importance of these nutrients for growth and TAG accumulation of *R. opacus*. As can be seen in Figure 1, the diminished concentrations of CaCl_2_ and MgSO_4_ (to 20% of the standard concentration), as of iron and the trace element solution (to 10% of the standard concentration) have only a minor effect on the final OD and the fatty acid contents. The limitation of the macroelement nitrogen (to 20% of the standard concentration) led to a drastically reduced growth but normal fatty acid contents. Contrary, the phosphorus limitation (to 20% of the standard concentration) resulted in an even higher final OD and fatty acid contents.

### Sucrose concentration

**Figure 2:**
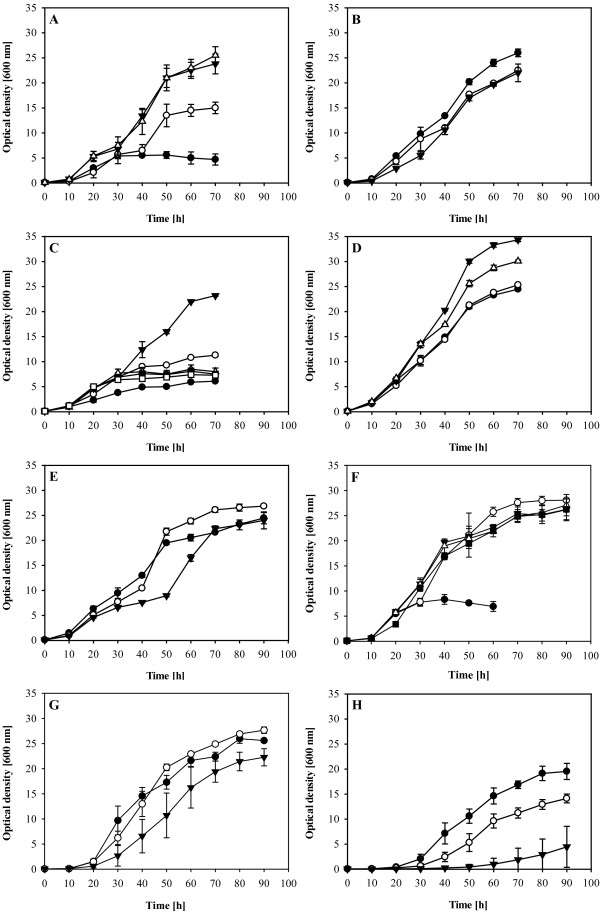
**Growth of *R. opacus *under different nutrient concentrations and pH values.***R. opacus* was grown in minimal medium as described in the material and methods section. The varied conditions were **A**: **●** 5 g l^-1^ sucrose, **○** 10 g l^-1^ sucrose, **▼** 20 g l^-1^ sucrose, Δ 30 g l^-1^ sucrose **B**: **●** 40 g l^-1^ sucrose, **○** 120 g l^-1^ sucrose, **▼** 240 g l^-1^ sucrose **C: ●** 0.1 g l^-1^ ammonium, **○** 0.2 g l^-1^ ammonium, **▼** 0.4 g l^-1^ ammonium, Δ 0.7 g l^-1^ ammonium, **■** 1.0 g l^-1^ ammonium, **□** 1.4 g l^-1^ ammonium **D: ●** 0.02 g l^-1^ magnesium, **○** 0.12 g l^-1^ magnesium, **▼** 0.51 g l^-1^ magnesium, Δ 1.01 g l^-1^ magnesium **E**: **●** 3.4 g l^-1^ phosphate , **○** 6.8 g l^-1^ phosphate, **▼** 13.6 g l^-1^ phosphate. **F**: The cultivations were started at an initial pH value of **●** 6.8, **○** 7.1, **▼** 7.4, Δ 7.7, **■** 8.0 **G**: Addition of NaCl to get an osmotic concentration of **●** 230 mOsm l^-1^, **○** 450 mOsm l^-1^, **▼** 800 mOsm l^-1^ **H**: Addition of NaCl to reach an osmolarity of **●** 1200 mOsm l^-1^, **○** 1600 mOsm l^-1^, **▼** 2000 mOsm l^-1^. Error bars represent the deviations of at least two independent cultivations.

**Figure 3:**
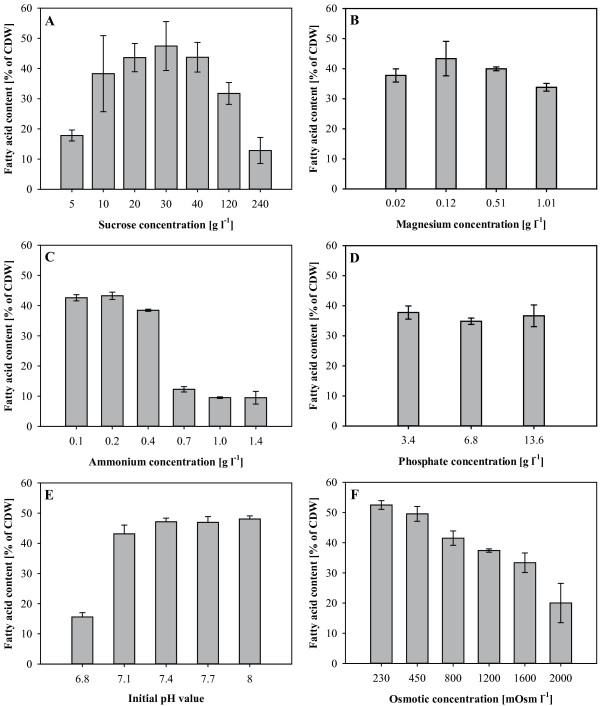
**Fatty acid contents of cells of *R. opacus *under different nutrient conditions.** Cells of *R. opacus* were grown in mineral salt medium and different concentrations of sucrose **(A)**, magnesium **(B)**, ammonium **(C)** or phosphate **(D)**, or with different initial pH values **(E)** or a different osmolarity of the medium **(F)**. After the growth experiment the fatty acid contents of the cell dry masses were determined. Error bars represent the deviations of at least two independent cultivations.

Figures 2A and B show the growth of *R. opacus* in presence of different sucrose concentrations. Best growth was obtained at concentrations of 20 to 40 g l^-1^, while lower concentrations were clearly not sufficient to obtain an OD higher than 20. It is striking, that sucrose concentrations up to 240 g l^-1^ inhibited growth only slightly (Figure 2B). However, in presence of these extremely high concentrations of sucrose the cells accumulated much less lipids as can be seen in Figure 3A. With 240 g l^-1^ sucrose only 13.5% of the cellular dry weight consisted of fatty acids, whereas in presence of 20 to 40 g l^-1^ sucrose fatty acid contents amounted up to 45.1%.

### Ammonium concentration

A crucial factor for an efficient TAG production is the concentration of ammonium in the fermentation medium. According to Figure 2C, the initial growth of *R. opacus* in Erlenmeyer flasks is more or less identical for all tested concentrations of ammonium, up to 1.4 g l^-1^. At low ammonium concentrations, exponential growth of the cultures stopped very early, indicating a depletion of the nitrogen source. Best growth was observed at a concentration of 0.4 g l^-1^ ammonium; under these conditions the final fatty acid content was 38.5% (Figure 3C). Lower ammonium concentrations provided slightly higher fatty acid contents, but a clearly reduced final OD. Higher concentrations of ammonium were not suitable under these conditions, leading to lower OD and fatty acid contents. Measurements of the ammonium concentrations confirmed that in cultures with up to 0.4 g l^-1^ ammonium, the nitrogen source was completely consumed, whereas in cultures with a higher initial ammonium concentration it was still available at the end of the experiment.

### Magnesium concentration

The influence of enhanced levels of MgSO_4_ on cell growth and lipid accumulation was investigated since magnesium ions are important cofactors. Higher magnesium concentrations might promote the accumulation of TAG, by enhancing the activity of the acetyl-CoA carboxylase. Figure 2D shows that *R. opacus* could achieve an about 35% higher OD, when cultivated in the presence of 0.51 g l^-1^ magnesium. Even in presence of 1.01 g l^-1^, a positive effect on the cell growth was observed. However, the lipid content of the cells on 1.01 g l^-1^ was slightly reduced in comparison to the control (Figure 3B). In an additional experiment the concentration of magnesium ions was increased by the addition of MgCl_2_ instead of MgSO_4_ (data not shown). Both, cell growth and the fatty acid content, were very similar to the experiment with MgSO_4_, yielding a maximal optical density with 0.51 g l^-1^ and a maximum in fatty acid synthesis with 0.12 g l ^1^ magnesium, added as MgCl_2_.

### Phosphate concentration

During the limitation experiments a positive effect on growth and fatty acid accumulation has been shown for 5-times reduced phosphate concentrations. Higher concentrations of phosphate (6.8 and 13.6 g l^-1^ phosphate) still enable fatty acid synthesis, of amounts that are comparably high as in the basic medium (Figure 3D). However, in the cultures with the highest amount of phosphate, a long *lag*-phase was observed which ranged from 30 and 50 hours of cultivation (Figure 2E).

### pH value

Growth and TAG storage of *R. opacus* were similar at initial pH values between 7.1 and at least 8. A lower initial pH impaired both, the final OD and the fatty acid content (Figures 2F and 3E). A measurement of the pH values after 32 hours showed a strong decrease, with a pH of 4.2 in the case of the culture that was started at pH 7.1 and pH values of 5.3, 6.2, 6.4 and 6.6 for the other cultures. The latter ones showed a typical growth and storage behavior in the following hours.

### Osmolarity

Concerning the tolerance of osmotic pressure, *R. opacus* showed good growth and fatty acid synthesis in liquid media with up to 1600 mOsm l^-1^, but the final OD as well as the final fatty acid content decreased successively in growth media with osmolarities higher than 450 mOsm l^-1^ (Figures 2G, H and 3F). At an osmotic concentration above 1600 mOsm l^-1^, only very slow growth occurred. In all cultures the initial *lag*-phase was shorter than 10 hours and the differences in the growth behavior result solely from different growth rates

## Discussion

The experiments concerning the sucrose concentration showed best growth and fatty acid production in a range of 20 to 40 g l^-1^ (Figures 2A and B). Lower as well as higher concentrations led in flask experiments to a decrease of accumulation. The culture that was grown in presence of 240 g l^-1^ sucrose stored even less fatty acids than the culture grown in presence of 5 g l^-1^ though at the latter concentration all carbon was depleted during the exponential growth phase. Due to the very low growth inhibition of sucrose even at concentrations as high as 240 g l^-1^, it seems unlikely that the bacterial metabolism is strongly affected. Moreover, the growth behavior of the culture containing 240 g l^-1^ sucrose (840 mOsm l^-1^ of the complete medium) is very similar to the growth in medium with an osmotic concentration of 800 mOsm l^-1^ (compare Figures 2B and G), so that the negative effect of high sucrose concentrations seems to be due to the enhanced osmotic pressure. Tolerance towards comparably high sucrose concentrations has also been shown in other organisms, for example in *Bacillus* sp. or in *Saccharomyces cerevisiae* (Belghith et al. 2012;Ando et al. 2006).

The tolerance of high sucrose concentrations is interesting also with regard to the report of (Kurosawa et al. 2010), who showed that the tolerance towards high glucose concentrations was paralleled by a long *lag*-phase, which could be reduced only by larger sizes of the inocula. A possible explanation for this different behavior is that sucrose belongs to the compatible solutes, while glucose potentially interferes with the bacterial metabolism. Additionally, the osmotic concentration of the defined medium with 240 g l^-1^ glucose and 13.4 g l^-1^ (NH_4_)_2_SO_4_ (Kurosawa et al. Kurosawa et al. 2010) amounts to 1600 mOsm l^-1^ and according to our data is at the limit of an acceptable growth rate.

Altering the ammonium concentration in flask experiments caused strong differences in the final OD. However, in the first 30 hours of cultivation only the cultures with ammonium concentrations lower than 0.4 g l^-1^ showed reduced growth, while concentrations of up to 1.4 g l^-1^ seemed not to be growth limiting (Figure 2C). Concerning the final fatty acid concentration there is a clear difference between the cultures that contain up to 0.4 g l^-1^ ammonium and the cultures with higher concentrations. This difference is easily explained by the fact that the cultures with low fatty acid concentration have not consumed the nitrogen in the medium, and consequently the fatty acid accumulation as TAG was not induced. In the cultures with a reduced ammonium concentration, the final OD correlated with the ammonium concentration (Figures 1 and 2C). With 0.4 g l^-1^, an OD of 24 was reached, while 0.2 g l^-1^ and 0.1 g l^-1^ led to final ODs of about 12 and 6. Growth with only 20% of the standard concentration of ammonium (0.07 g l^-1^, provided as 0.2 g l^-1^ NH_4_Cl) gave a final OD of 20% of the control experiment. Thus it seems reasonable that the differences in the final OD can be explained either by a reduced cell growth, caused by too low ammonium concentrations, with normal fatty acid production or by a maximal cell growth but without a significant fatty acid production in the case of ammonium concentrations higher than 0.4 g l^-1^.

It was shown that the final OD of *R. opacus* was positively affected by high concentrations of both, MgSO_4_ or MgCl_2_ (Figure 2D). In comparison with the good growth and fatty acid synthesis of *R. opacus* under reduced MgSO_4_ concentrations (Figure 1), it is obvious that the sulfate concentration (0.08 g l^-1^ in the basic medium and 0.02 g l^-1^ under reduced conditions) is not limiting in the flask experiments and the higher OD with additional MgSO_4_ is caused by the magnesium ions. The fatty acid content and profile were not significantly altered, but the accumulation appeared to be enhanced in the presence of 0.12 g l^-1^ and 0.51 g l^-1^ magnesium and slightly decreased in the presence of higher magnesium concentrations in the culture medium. A continued cell division of *R. opacus* during the nitrogen limitation can be ruled out since the lack of nitrogen limits the DNA replication and protein biosynthesis. However, it is possible that the higher OD reflects a continued cell elongation in presence of enhanced magnesium concentrations, which might be due to an ongoing membrane synthesis.

The small differences in TAG storage between the control and 5-times reduced or up to 50-times increased magnesium concentrations indicate that the availability of magnesium ions as cofactor for fatty acid synthesis was sufficient under all tested conditions or had only a minor effect. The osmotic concentration of the growth medium in these experiments was in the range of 253 to 376 mOsm l^-1^ and thus should not be the cause for the observed differences in final OD and fatty acid content (compare with Figure 2G).

In the limitation experiments, a reduction of the phosphate concentration led to a 20% increase in OD and a 10% increase in fatty acid content. Since a reduction of the phosphate concentration severely decreased the buffer capacity of the medium, the pH was manually adjusted to that of the control culture. Without this pH adjustment, the final OD and fatty acid content were very low and similar to a culture with an initial pH of 6.8 (Figure 2F), which is probably due to a rapid decrease of the pH value (data not shown). In the following experiments, enhanced levels of phosphate (2- and 4-times of the standard concentration) were investigated to enhance the buffer capacity, but were not found to be suitable to improve cell growth or fatty acid accumulation. In fact, the growth rate in the first 40 hours was reduced, and the onset of TAG storage (marked by a second increase in OD between 40 and 70 hours; see Figure 2E) was delayed in the cultures with the highest phosphate concentration. Since the osmotic concentration of the cultures with 13.6 g l^-1^ phosphate (450 mOsm l^-1^) is about the same as in the cultures with 120 g l^-1^ sucrose concentration (488 mOsm l^-1^; Figure 2B) and the cultures containing NaCl to get an osmotic concentration of 450 mOsm l^-1^ (Figure 2G), the delay in growth should not be caused by the enhanced osmotic concentration.

A possible reason for the slower growth rate and delay in accumulation could be a regulatory role of the phosphate ions. In *E. coli* it has been shown that phosphate concentrations above 37 mM (equal to 3.5 g l^-1^) trigger the maintenance of cellular viability in the stationary phase (Schurig-Briccio et al. 2008), and in *Clostridium perfringens* the phosphate concentration influences the onset of sporulation (Philippe et al. 2006). In *R. opacus* the intracellular phosphate concentration could be involved in determining the energy status and thus inhibit the TAG synthesis.

Flask experiments with different initial pH values have shown that a good fatty acid production is possible when the initial pH is in the range of 7.1 to 8 (Figure 3E). In the first 32 hours, a strong decrease of the pH was observed, and the main fatty acid biosynthesis in the flask experiments seems to occur at a pH higher than 5. Lower pH values, as in the culture with an initial pH of 6.8 are not suitable for a continued growth or fatty acid accumulation (Figures 2F and 3E).

Overall this study provided further insights in the nutritional requirements of the lipid producing bacterium *R. opacus*. The results can be used for the optimization of the fermentation process in stirred tank reactors with the aim to produce high amounts of fatty acids as an alternative source for biodiesel or renewable diesel. In this regard the high salt tolerance is of importance, since the addition of strong acids or bases for a pH control during fermentation leads to an increase of the osmotic pressure of the medium.

## Competing interests

The authors declare that they have no competing interests.

## References

[B1] Adamczak M Bornscheuer UT Bednarski W The application of biotechnological methods for the synthesis of biodiesel Eur J Lipid Sci Technol 2009 3 808 813

[B2] Alvarez HM Steinbüchel A Triacylglycerols in prokaryotic microorganisms Appl Microbiol Biotechnol 2002 3 367 376 10.1007/s00253-002-1135-0 12466875

[B3] Alvarez HM Mayer F Fabritius D Steinbüchel A Formation of intracytoplasmic lipid inclusions by *Rhodococcus opacus* strain PD630 Arch Microbiol 1996 3 377 386 10.1007/s002030050341 8661931

[B4] Al-Zuhair S Production of biodiesel: possibilities and challenges Biofuels, Bioprod Biorefin 2007 3 57 66 10.1002/bbb.2

[B5] Ando A Tanaka F Murata Y Takagi H Shima J Identification and classification of genes required for tolerance to high-sucrose stress revealed by genome-wide screening of *Saccharomyces cerevisiae* FEMS Yeast Res 2006 3 249 267 10.1111/j.1567-1364.2006.00035.x 16487347

[B6] Antoni D Zverlov VV Schwarz WH Biofuels from microbes Appl Microbiol Biotechnol 2007 3 23 35 10.1007/s00253-007-1163-x 17891391

[B7] Belghith KS Dahech I Belghith H Mejdoub H Microbial production of levansucrase for synthesis of fructooligosaccharides and levan Int J Biol Macromol 2012 3 451 458 10.1016/j.ijbiomac.2011.12.033 22234294

[B8] Brandl H Goss RA Lenz RW Fuller RC *Pseudomonas oleovorans* as a source of poly(β-hydroxyalkanoates) for potential applications as biodegradable polyesters Appl Environ Microbiol 1988 3 1977 1982 16347708 10.1128/aem.54.8.1977-1982.1988 PMC202789

[B9] Cronan JE Jr Waldrop GL Multi-subunit acetyl-CoA carboxylases Prog Lipid Res 2002 3 407 435 10.1016/S0163-7827(02)00007-3 12121720

[B10] Finnerty WR The biology and genetics of the genus *Rhodococcus* Annu Rev Microbiol 1992 3 193 218 10.1146/annurev.mi.46.100192.001205 1444254

[B11] Hamada T Maeda Y Matsuda H Sameshima Y Honda K Omasa T Kato J Ohtake H Effect of cell-surface hydrophobicity on bacterial conversion of water-immiscible chemicals in two-liquid-phase culture systems J Biosci Bioeng 2009 3 116 120 10.1016/j.jbiosc.2009.03.009 19619857

[B12] Hori K Kobayashi A Ikeda H Unno H *Rhodococcus aetherivorans* IAR1, a new bacterial strain synthesizing poly(3-hydroxybutryrate-*co*-3-hydroxyvalerate) from toluene J Biosci Bioeng 2009 3 145 150 10.1016/j.jbiosc.2008.10.005 19217552

[B13] Kurosawa K Boccazzi P De Almeida NM Sinskey AJ High-cell-density batch fermentation of *Rhodococcus opacus* PD630 using a high glucose concentration for triacylglycerol production J Biotechnol 2010 3 212 218 10.1016/j.jbiotec.2010.04.003 20412824

[B14] Lang S Philip JC Surface-active lipids in *Rhodococci* Antonie Van Leeuwenhoek 1998 3 59 70 10.1023/A:1001799711799 10068789

[B15] Luque R Herrero-Davila L Campelo JM Clark JH Hidalgo JM Luna D Marinas JM Romero AA Biofuels: A technological perspective Energy Environ Sci 2008 3 542 564 10.1039/b807094f

[B16] McDonald RI Fargione J Kiesecker J Miller WM Powell J Energy sprawl or energy efficiency: Climate policy impacts on natural habitat for the United States of America PLoS One 2009 3 e6802 10.1371/journal.pone.0006802 19707570 PMC2728545

[B17] Na K-S Kuroda A Takiguchi N Ikeda T Ohtake H Kato J Isolation and characterization of benzene-tolerant *Rhodococcus opacus* strains J Biosci Bioeng 2005 3 378 382 10.1263/jbb.99.378 16233805

[B18] Pfennig N *Rhodopseudomonas globiformis* sp. n. a new species of the *Rhodospirillaceae* Arch Microbiol 1974 3 197 106 10.1007/BF00446317

[B19] Philippe VA Méndez MB Huang IH Orsaria LM Sarker MR Grau RR Inorganic phosphate induces spore morphogenesis and enterotoxin production in the intestinal pathogen *Clostridium perfringens* Infect Immun 2006 3 3651 3656 10.1128/IAI.02090-05 16714597 PMC1479234

[B20] Ratledge C Monograph of single cell oils 2010 Urbana, USA: ACS Press 67 90

[B21] Röttig A Wenning L Bröker D Steinbüchel A Fatty acid alkyl esters: perspectives for production of alternative biofuels Appl Microbiol Biotechnol 2010 3 1713 1733 10.1007/s00253-009-2383-z 20033403

[B22] Rude MA Schirmer A New microbial fuels: a biotech perspective Curr Opin Microbiol 2009 3 274 281 10.1016/j.mib.2009.04.004 19447673

[B23] Schlegel HG Kaltwasser H Gottschalk G Ein submersverfahren zur kultur wasserstoff oxidierender bakterien: wachstumsphysiologische untersuchungen Arch Mikrobiol 1961 3 209 22213747777

[B24] Schurig-Briccio LA Rintoul MR Volentini SI Farías RN Baldomà L Badía J Rodríguez-Montelongo RVA A critical phosphate concentration in the stationary phase maintains *ndh* gene expression and aerobic respiratory chain activity in *Escherichia coli* FEMS Microbiol Lett 2008 3 76 83 10.1111/j.1574-6968.2008.01188.x 18492062

[B25] Uthoff S Bröker D Steinbüchel A Current state and perspective of producing biodiesel-like compounds by biotechnology Microb Biotechnol 2009 3 551 565 10.1111/j.1751-7915.2009.00139.x 21255288 PMC3815363

[B26] Voss I Steinbüchel A High cell density cultivation of *Rhodococcus opacus* for lipid production at a pilot-plant scale Appl Microbiol Biotechnol 2001 3 547 555 10.1007/s002530000576 11414319

[B27] Wältermann M Luftmann H Baumeister D Kalscheuer R Steinbüchel A *Rhodococcus opacus* strain PD630 as a new source of high-value singel-cell oil? Isolation and characterization of triacylglycerols and other storage lipids Microbiology (SGM) 2000 3 1143 1149 10.1099/00221287-146-5-1143 10832641

